# Temporal, Location- and Symptom-Specific Likelihood of Patient-Reported Sensory Symptoms Related to Oxaliplatin-Induced Peripheral Neuropathy (OIPN) in Patients Receiving Oxaliplatin for Three Months

**DOI:** 10.3390/cancers14051212

**Published:** 2022-02-25

**Authors:** David Zahrieh, Daniel Satele, Ellen M. Lavoie Smith, Charles L. Loprinzi, Jennifer Le-Rademacher

**Affiliations:** 1 Department of Quantitative Health Sciences, Mayo Clinic, Rochester, MN 55905, USA; zahrieh.david@mayo.edu (D.Z.); satele.daniel@mayo.edu (D.S.); 2 Department of Acute, Chronic, and Continuing Care, University of Alabama at Birmingham School of Nursing, Birmingham, AL 35294, USA; esmith3@uab.edu; 3 Department of Medical Oncology, Mayo Clinic, Rochester, MN 55905, USA; cloprinzi@mayo.edu

**Keywords:** chemotherapy-induced peripheral neuropathy, clinical trials, colorectal cancer, statistical modeling, symptom intervention

## Abstract

**Simple Summary:**

There are no known effective preventative interventions for oxaliplatin-induced peripheral neuropathy (OIPN) sensory symptoms of numbness, tingling and pain other than limiting drug exposure. With the shorter 3-month duration of oxaliplatin increasingly being used, compared to the previous 6-month standard, we were motivated to quantify the temporal, location- and symptom-specific likelihood of patient-reported sensory symptoms related to OIPN in 141 patients from the placebo arms of two multisite OIPN prevention trials exposed to oxaliplatin for 3 months. Despite a shorter duration of oxaliplatin, we show that OIPN was still pervasive, with patients experiencing considerable mild to moderate numbness and tingling in the lower and upper distal extremities. To avoid the debilitating sequelae from OIPN and to ensure that patients continue to receive the most efficacious doses of oxaliplatin, identification of effective OIPN preventative interventions is still needed, regardless of whether oxaliplatin is planned to be given for 3 versus 6 months.

**Abstract:**

While oxaliplatin-induced peripheral neuropathy (OIPN) is more common and severe in patients who receive the previous standard, 6-month oxaliplatin-based treatment, we hypothesized that OIPN was still pervasive in patients who received shorter, 3-month-treatment regimens. Using six EORTC QLQ-CIPN20 questions that quantify numbness (N), tingling (T) and shooting/burning pain (P) in upper/lower distal extremities, our aim is to quantify patient-reported responses over 3 months (6 cycles) of oxaliplatin regarding symptom-specific timing, location and severity. For each question, patients were asked how each of the sensory symptoms had affected them during the preceding week, with 1 = “Not at all”, 2 = “A little”, 3 = “Quite a bit” and 4 = “Very much”. The proportional odds model for the cumulative log odds of response that allowed symptom-specific patient heterogeneity to be obtained was applied to a pooled dataset from the placebo arms of two multisite OIPN prevention trials and fit separately to the upper/lower distal extremities. For each symptom, we report the cycle-specific marginal probabilities for each response. In 141 patients, substantial patient heterogeneity in the likelihood, at a given cycle, of a more severe response for a symptom was present. Distinct patterns in the probabilities for each response over time for N and T were observed between the upper/lower distal extremities, while the probabilities of a response >1 for P was largely negligible in both locations. Despite the decrease in exposure to oxaliplatin from 6 to 3 months, OIPN was still pervasive with patients experiencing considerable N and T in the fingers (or hands) and toes (or feet).

## 1. Introduction

Oxaliplatin is a key component of the FOLFOX (85 mg/m^2^ every two weeks) and CAPOX (130 mg/m^2^ every three weeks) multi-drug regimens that are used to treat colorectal cancers. However, neuropathy is a common and troublesome long-term side effect of oxaliplatin. In total, 85–95% of patients receiving oxaliplatin experience oxaliplatin-induced peripheral neuropathy (OIPN) [1]. No known effective agents are recommended for the prevention of OIPN. There are numerous characteristics of OPIN, including numbness (N), tingling (T) and shooting/burning pain (P), which spread proximally to affect both lower and upper extremities.

The results of the SCOT trial [2], which is the largest single randomized study regarding the adjuvant treatment of colorectal cancer, recently demonstrated the non-inferiority of a shorter duration (3 months as opposed to 6 months) of either of the two adjuvant chemotherapy regimens for patients with high-risk stage II and stage III cancer of the colon and rectum. This finding was consistent with the meta-analysis of six worldwide studies conducted by the IDEA collaboration and, consequently, the recommended number of FOLFOX and CAPOX treatment cycles for adjuvant colorectal cancer has recently changed from 6 months to 3 months, particularly for those low-risk patients with T1-3, N1 disease [3,4]. Further, the SCOT trial reported that patients, both high- and low-risk, treated with adjuvant therapy for the shorter 3-month duration had substantially lower rates of neuropathy—N, T and P in their hands and feet. These data support the use of shorter 3-month oxaliplatin treatment courses.

Despite the decrease in total cycles and cumulative oxaliplatin dosage, some patients still experience dose-limiting oxaliplatin OIPN. In our Ca/Mg neuropathy prevention trial, for instance, where 94% of the 353 enrolled patients presented with T1-3 disease and received 6 months (12 cycles) of FOLFOX, the median cycle number when patients experienced dose-limiting OIPN was cycle 6 (3 months) and only about 60% were able to receive full-dose oxaliplatin by this time point; this serves as an indirect measure of OIPN, as most patients who stop receiving full-dose oxaliplatin do so due to OIPN [5]. Because the cumulative doses of oxaliplatin are similar between the 3-month FOLFOX regimen (85 mg/m^2^ × 6 doses) and the multi-drug 3-month CAPOX regimen (130 mg/m^2^ × 4 doses), they are expected to have a similar effect on dose-limiting oxaliplatin OIPN. From these data, we hypothesized that, while OIPN is more common and severe in patients who receive 6-month oxaliplatin-based treatment, OIPN is still pervasive in patients who receive shorter 3-month treatment regimens.

The aim of the current research study, therefore, is two-fold. First, using pooled data from the placebo arms of two completed OIPN prevention clinical trials of patients receiving FOLFOX for colon cancer, our aim is to develop and evaluate a statistical model that is compatible with the complex clinical course of OIPN during 3 months (6 cycles) of oxaliplatin to quantify the likelihood of symptom-specific timing, location and severity of patient-reported responses of N, T and P in the upper/lower distal extremities. Second, our aim is to illustrate how this information could directly inform the study design of subsequent placebo-controlled OIPN symptom intervention trials, whereby patients receive the shorter 3-month duration (6 cycles) of oxaliplatin.

To address these two aims, this article is organized as follows: In Section 2, we describe the data used to support the first aim. Herein, we define the statistical model and detail the analytic approach. Then, we present the results, in Section 3, obtained by applying the statistical model separately to the upper/lower distal extremities. In Section 4, we address the second aim. Herein, we illustrate how the results obtained from addressing the first aim can be used to design a two-arm, randomized, placebo-controlled OIPN study to determine whether an experimental intervention is effective at preventing OIPN compared with placebo in patients receiving the shorter, 3-month duration (6 cycles) of oxaliplatin. In Section 5, we conclude our presentation by summarizing our research study and discussing additional points of consideration in the design and analysis of such trials.

## 2. Materials and Methods

### 2.1. Data

The current study uses data from two completed randomized double-blind placebo-controlled trials conducted to evaluate potential therapy for prevention of oxaliplatin-induced neuropathy (North Central Cancer Treatment Group [NCCTG] N08CB; MC11C4) [5,6]. In MC11C4, 50 patients, scheduled to undergo oxaliplatin-based therapy (FOLFOX) for stages II–III (67%) or stage IV (33%) colon cancer, were randomized to receive venlafaxine or placebo through their last dose of oxaliplatin. In NCCTG N08CB, 353 patients with stages II–III (94%) or stage IV (6%) colon cancer undergoing adjuvant therapy with FOLFOX were randomly assigned to intravenous calcium/magnesium before and after oxaliplatin, a placebo before and after, or calcium/magnesium before and placebo after. Neither study supported the use of the interventional agents for preventing OIPN in clinical practice. Because aim 1 of the current research study is to quantify the temporal, location- and symptom-specific likelihood of patient-reported sensory symptoms related to OIPN in the natural history setting during the first 3 months (6 cycles) of oxaliplatin exposure, we focused exclusively on the placebo arms in studies MC11C4 and NCCTG N08CB and on the first 3 months (6 cycles) of FOLFOX treatment. It is important to note that all patients included in this analysis received oxaliplatin for treatment of colorectal cancer while receiving placebo for prevention of OIPN.

Patient-reported responses from the 6 European Organization of Research and Treatment of Cancer (EORTC) Quality of Life Questionnaire—Chemotherapy-Induced Peripheral Neuropathy (QLQ-CIPN20) questions that quantify N, T and P in upper/lower distal extremities (Table S1; Q31–36) were recorded on day 1 of each 2-week cycle prior to oxaliplatin treatment (cycles 1–6), as well as 2 weeks after the 6th cycle (day 1 of cycle 7, prior to oxaliplatin treatment) in the placebo arms of the two recently completed OIPN prevention trials. This means that cycle 1 served as a baseline (prior to oxaliplatin exposure), while patient-reported responses measured at cycles 2–6 and at 2 weeks post-cycle 6 served as the post-baseline measurements. For each question, patients were asked how each of the sensory symptoms had affected them during the preceding week, with 1 = “Not at all”, 2 = “A little”, 3 = “Quite a bit” and 4 = “Very much”. For a single patient, the format of the ordinal data in the fingers (or hands) is shown in Table 1A. For the 3 EORTC QLQ-CIPN20 questions that quantify N, T and P in the toes (or feet), the format of the ordinal data is the same.

### 2.2. Model

Wolf and colleagues (2012) [7] recommended that symptoms in the lower extremities should be modeled distinctly from those in the upper extremities. Following this recommendation, two separate models were assumed, one for fingers (or hands) (Model 1) and one for the toes (or feet) (Model 2). Because interest lied in characterizing effects that referred both to the 4 individual response categories, as well as grouping of response categories, a proportional odds model for the cumulative log odds of response that allowed symptom-specific patient heterogeneity to be obtained was applied to the pooled dataset from the placebo arms of the two OIPN prevention trials and fit separately to the distal extremities. Due to data sparsity, however, response outcomes of 3–4 (“Quite a bit” and “Very much”) were combined, such that there were K=3 response categories and K−1=2 cumulative logits. Using the notation detailed in Table 1B, we can express Model 1 (and similarly for Model 2) as the log odds of being greater than or equal to a particular response category and we can write


$$\mathrm{logit}\left[P\left(y_{its}\geq k\text{|}u_{is}\right)\right]=\log\left[\frac{P\left(y_{its}\geq k\right)}{1-P\left(y_{its}\geq k\right)}\right]\;\;=\alpha_{k}+\beta_{1}N+\beta_{2}T+\beta_{3}T2+\beta_{4}T3+\beta_{5}T4+\beta_{6}T5+\beta_{7}T6+\beta_{8}T7\;+\beta_{9}N\;*T2+\beta_{10}N*T3\;+\beta_{11}N*T4+\beta_{12}N*T5+\beta_{13}N*T6+\beta_{14}N*T7\;\;+\beta_{15}T*T2+\beta_{16}T*T3+\beta_{17}T*T4\;+\beta_{18}T*T5+\beta_{19}T*T6+\beta_{20}T*T7\;+u_{is},\;k=2,3.$$

In this generalized linear mixed model for the cumulative logits, $\log\left[\frac{P\left(y_{its}\geq k\right)}{1-P\left(y_{its}\geq k\right)}\right]$ describes the log odds of two cumulative probabilities and measures how likely the response is to be in category k or higher versus in a category lower than *k*. Specifically, one of the two cumulative logits describes the log odds of responding “Quite a bit”/“Very much” (y=3) versus “Not at all” or “A little” (y∈1,2), while the other cumulative logit describes the log odds of responding “A little” or “Quite a bit”/“Very much” (y∈2,3) versus “Not at all” (y=1). Because of the proportional odds assumption, the effect, as measured by the difference in log odds, is constrained to be the same for each cumulative logit (y=3 vs. y∈1,2 and y∈2,3 vs. y=1). Notably, the model has patient effects for each symptom. The patient effects $\left(u_{i1},\;u_{i2},u_{i3}\right)$ is a multivariate random effect that describes patient heterogeneity for N, T and P.

### 2.3. Analysis

To evaluate the appropriateness of the proportional odds assumption in Models 1 and 2, we also fit the corresponding non-proportional odds model and applied the Bayesian information criterion (BIC) to confirm that the BIC expressed a preference for the more parsimonious proportional odds model (data not shown). Further, the BIC was applied to evaluate the variance–covariance structure of each model.

Multiple imputation was applied to handle missing responses [8]. We know that oxaliplatin dose (mg) is both predictive of the probability of missingness and predictive of the responses. However, we had no interest in making inference on the oxaliplatin dose or conditional upon the oxaliplatin dose; in other words, oxaliplatin dose was treated as an extraneous variable, but incorporated into the imputation model because the inclusion of an extraneous variable that is highly correlated with the response greatly improves the imputations. Given the extraneous variable, the missing-at-random assumption became defensible; therefore, multiple imputation was applied to handle missing responses and the multiple imputation model included the extraneous variable, but, otherwise, was compatible with the analysis model.

For each symptom, we report the estimated difference in log odds of a more severe response between each cycle (cycles 2–6 and, at 2 weeks, post-cycle 6) and cycle 1 (day 1 of cycle 1, prior to oxaliplatin treatment) along with the corresponding 95% confidence interval (CI). Additionally, for each symptom, we report the cycle-specific marginal or population-averaged probabilities (averaged over patients) for each response outcome and the corresponding 95% CI.

The SAS procedure PROC GLIMMIX was used to fit both Model 1 and Model 2 using adaptive Gauss–Hermite quadrature to approximate the likelihood function in obtaining maximum likelihood estimates. The number of quadrature points were chosen based on monitoring the convergence of estimates and standard errors. The SAS procedure PROC MI was used to perform multiple imputation with 50 imputed datasets assuming an arbitrary missing-data pattern with the fully conditional specification method.

## 3. Results

One hundred forty-one patients scheduled to undergo oxaliplatin-based therapy (FOLFOX) for stages II–III (87%) or stage IV (13%) colon cancer were randomized to the placebo arm in OIPN prevention trials NCCTG N08CB (N = 118) and MC11C4 (N = 23), thus included in these analyses. Among the 141 patients analyzed, the median age (range indicated in squared brackets) in years was 56.0 [28.0, 83.0], 51% were female and 89% were Caucasian. On day 1 of cycle 1, prior to oxaliplatin treatment cycle 1, the number (%) of patients who reported “Not at all” (y=1) in both the upper and lower extremities for N, T and P was 121 (88%), 121 (88%) and 134 (97%), respectively. Two weeks after cycle 6, the number (%) of patients who reported “Not at all” (y=1) in both the upper and lower extremities for N, T and P was 54 (47%), 28 (24%) and 89 (77%), respectively.

### 3.1. Model 1—Fingers (or Hands)

At a given cycle, substantial patient heterogeneity in the likelihood of a more severe response for a symptom was present and the degree of heterogeneity was quite different between each symptom, with the largest degree of heterogeneity seen with P. The estimated variance components ${\hat{\sigma }}_{1}^{2}$ for $\left\{u_{i1}\right\}$, ${\hat{\sigma }}_{2}^{2}$ for $\left\{u_{i2}\right\}$ and ${\hat{\sigma }}_{3}^{2}$ for $\left\{u_{i3}\right\}$ corresponding to N, T and P were 4.55, 3.18 and 8.92, respectively; the estimated standard deviations were 2.13, 1.78 and 2.99. The large degree of heterogeneity implies tremendous variability in between-patient log odds of a more severe response at a given post-oxaliplatin cycle, compared with cycle 1. Further, concerning the estimated correlation, patient effects $\left(u_{i1},\;u_{i2},u_{i3}\right)$ were highly correlated (${\hat{\rho }}_{NT}=0.86,\;{\hat{\rho }}_{NP}=0.75,\;{\hat{\rho }}_{TP}=0.80$), with the largest correlation being between N and T.

**Figure 1:**
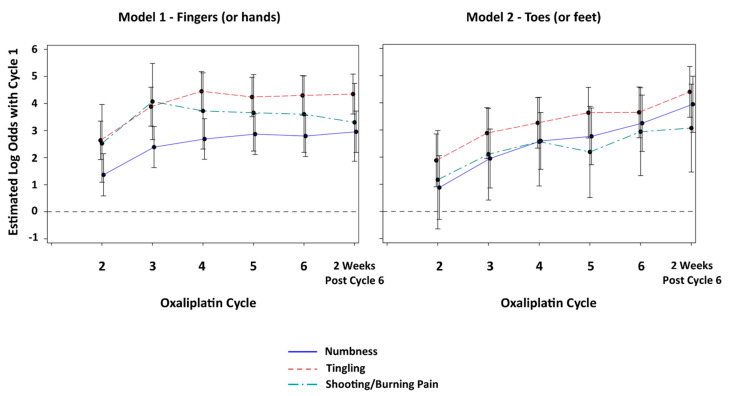
Estimated log odds of a more severe response at cycles 2, 3, 4, 5, 6 and at 2 weeks post-cycle 6 compared with cycle 1 (baseline) for numbness, tingling and shooting/burning pain in the fingers (or hands) (**LEFT**) and in the toes (or feet) (**RIGHT**).

Figure 1 (LEFT) graphically displays the estimated log odds of a more severe response at cycles 2, 3, 4, 5 and 6, as well as 2 weeks post-cycle 6, compared with cycle 1 for N, T and P. For N, the estimated log odds compared with cycle 1 increased in a somewhat linear fashion through 2 weeks post-cycle 6; however, the effect was considerably less marked than T. For T, the estimated log odds compared with cycle 1 peaked at cycle 4 and then plateaued. Due to the large degree of patient heterogeneity seen with P, the confidence intervals for the estimated log odds compared with cycle 1 were so wide that they precluded any meaningful interpretation.

**Figure 2:**
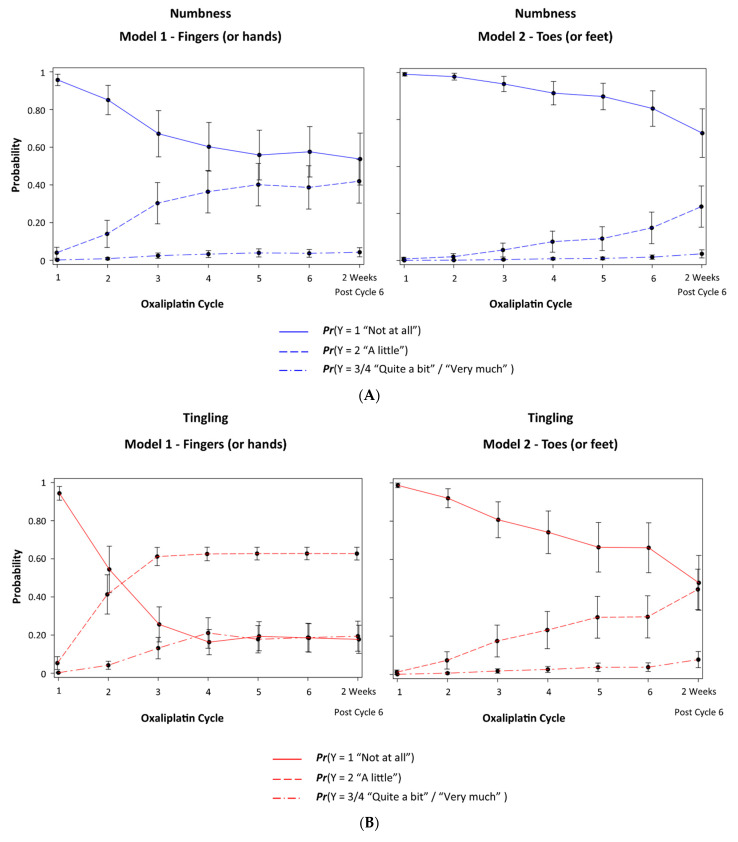

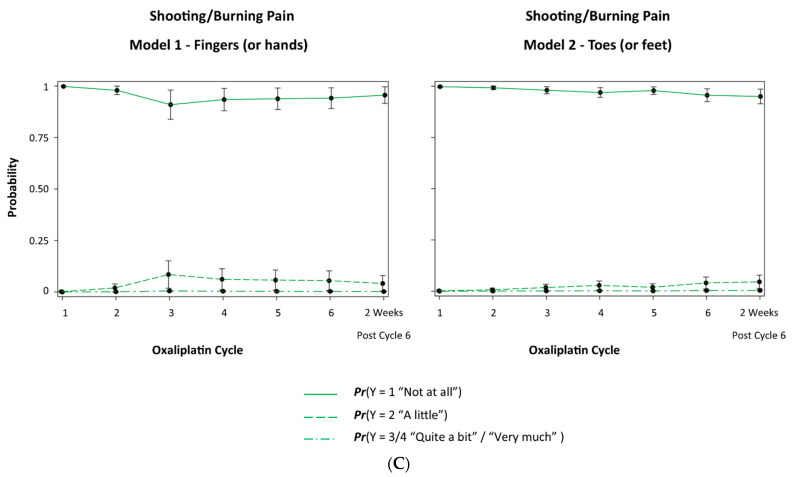
(**A**) Population-averaged probability (averaged over patients) of each ordinal response (Y)—numbness; (**B**) population-averaged probability (averaged over patients) of each ordinal response (Y)—tingling; (**C**) population-averaged probability (averaged over patients) of each ordinal response (Y)—shooting/burning pain—in the fingers (or hands) (LEFT) and in the toes (or feet) (RIGHT).

Distinct patterns in the population-averaged probabilities (averaged over patients) for each response over time for N and T were observed (Figure 2A (LEFT) and Figure 2B (LEFT)). Although the probability *Pr(Y = 1 “Not at all”)* decreased over time for N, the increase in the probability *Pr(Y = 2 “A little”)* never exceeded the probability *Pr(Y = 1 “Not at all”)* following 6 cycles. The probability *Pr(Y = 1 “Not at all”)* and *Pr(Y = 2 “A little”)* for N 2 weeks after cycle 6 was 0.535 (95% CI: 0.397, 0.674) and 0.422 (95% CI: 0.305, 0.539), respectively. Further, the probability *Pr(Y = 3/4 “Quite a bit”/“Very Much”)* for N never exceeded 0.10. Following 6 cycles of oxaliplatin, the probability *Pr(Y = 3/4 “Quite a bit”/“Very Much”)* for N was 0.043 (95% CI: 0.019, 0.066).

For T, however, there was a marked decrease in the probability *Pr(Y = 1 “Not at all”)*, which leveled off at a probability of approximately 0.20 by cycle 4. That marked decrease was replaced by a corresponding marked increase in the probability *Pr(Y = 2 “A little”)*, which leveled off at a probability of 0.60 by cycle 4. The probability *Pr(Y = 1 “Not at all”)* and *Pr(Y = 2 “A little”)* for T, 2 weeks after cycle 6, was 0.176 (95% CI: 0.102, 0.250) and 0.631 (95% CI: 0.598, 0.664), respectively. Additionally, the probability *Pr(Y = ¾ “Quite a bit”/“Very Much”)* for T increased to 0.20 by cycle 4 and then stabilized. Following 6 cycles of oxaliplatin, the probability *Pr(Y = ¾ “Quite a bit”/“Very Much”)* for T was 0.193 (95% CI: 0.114, 0.272).

The probabilities of a response >1 for P was largely negligible (Figure 2C [LEFT]). The probability *Pr(Y = 1 “Not at all”)*, *Pr(Y = 2 “A little”)* and *Pr(Y = ¾ “Quite a bit”/“Very Much”),* 2 weeks after cycle 6, was 0.956 (95% CI: 0.916, 0.997), 0.042 (95% CI: 0.003, 0.080) and 0.002 (95% CI: 0.000, 0.005), respectively.

### 3.2. Model 2—Toes (or Feet)

The BIC criterion expressed a preference for a model with a single patient-level random intercept $u_{i}$. The estimated variance of the random intercept was relatively large, ${{\hat{\sigma }}^{2}}_{\left(u\right)}=4.75$, indicating that, at a given cycle, patients were highly heterogeneous in the likelihood of reporting a more severe response.

Figure 1 (RIGHT) graphically displays the estimated log odds of a more severe response at cycles 2, 3, 4, 5 and 6, as well as 2 weeks after cycle 6, compared with cycle 1 for N, T and P. Compared with cycle 1, the estimated log odds generally showed a steady increase throughout 2 weeks post-cycle 6 for N and T. As seen with the fingers (or hands), the estimated log odds compared with cycle 1 for P were too imprecise to discern a pattern.

As with the fingers (or hands), distinct patterns in the population-averaged probabilities (averaged over patients) for each response over time for N and T were also observed in the toes (or feet) (Figure 2A (RIGHT) and Figure 2B (RIGHT)). The probability *Pr(Y = 1 “Not at all”)* decreased only modestly over time for N, such that, 2 weeks after cycle 6, the probability *Pr(Y = 1 “Not at all”)* was 0.680 (95% CI: 0.549, 0.811). The probability *Pr(Y = 2 “A little”)* for N increased only modestly, such that, 2 weeks after cycle 6, the probability *Pr(Y = 2 “A little”)* was 0.286 (95% CI: 0.174, 0.398). The probability *Pr(Y = 3/4 “Quite a bit”/“Very Much”)* for N never exceeded 0.05. Following 6 cycles of oxaliplatin, the probability *Pr(Y = 3/4 “Quite a bit”/“Very Much”)* for N was 0.034 (95% CI: 0.013, 0.055).

For T, however, there was a marked and steady decrease in the probability *Pr(Y = 1 “Not at all”)* and a marked and steady increase in the probability *Pr(Y = 2 “A little”)*, which came together 2 weeks after cycle 6. The probability *Pr(Y = 1 “Not at all”)* and *Pr(Y = 2 “A little”)* for T, 2 weeks after cycle 6, was 0.478 (95% CI: 0.333, 0.623) and 0.447 (95% CI: 0.338, 0.555), respectively. The probability *Pr(Y = 3/4 “Quite a bit”/“Very Much”)* for T increased to 0.075 (95% CI: 0.034, 0.117) 2 weeks after cycle 6.

As seen in the fingers (or hands), the probabilities of a response >1 for P in the toes (or feet) was largely negligible (Figure 2C (RIGHT)). The probability *Pr(Y = 1 “Not at all”)*, *Pr(Y = 2 “A little”)* and *Pr(Y = 3/4 “Quite a bit”/“Very Much”),* 2 weeks after cycle 6, was 0.951 (95% CI: 0.916, 0.987), 0.045 (95% CI: 0.013, 0.077) and 0.004 (95% CI: 0.001, 0.007), respectively.

## 4. Design Illustration

### 4.1. Materials and Methods

#### 4.1.1. Study Design

The proportional odds model fit these data well. To address aim 2, let us suppose now that we are interested in designing a two-arm, randomized, placebo-controlled OIPN study to determine whether an experimental intervention is effective at preventing OIPN compared with placebo in patients receiving 6 cycles of oxaliplatin. Here, the primary endpoint is the serially measured sensory scores for N, T and P in upper/lower extremities self-reported by the patient on day one of each cycle prior to oxaliplatin treatment (cycles 1–6) and then 2 weeks post-cycle 6. For the primary analysis, the same proportional odds model used in the data analysis of the pooled placebo arms (NCCTG N08CB; MC11C4) is adopted, one for the fingers (or hands) and one for the toes (or feet).

#### 4.1.2. Hypothesis

In such a study, the question of main scientific interest concerns the comparison of the two arms in terms of their patterns of change from the baseline of the odds of being greater than or equal to a particular response category for N, T and P in the upper (Model 1) and lower (Model 2) extremities. For each model, this comparison is made by testing an arm-by-time interaction, namely, the following null versus alternative hypotheses:

$H_{0}:$ No arm x time interaction versus.

$H_{a}:$ Patterns of change from baseline are not the same.

The test of the interaction is based on a multivariate Wald test statistic.

To operationalize the testing of the hypotheses, say, for the fingers (or hands), it is instructive to write out the form of Model 1 (which would be the same for Model 2). Using the same notation detailed in Table 1B, we introduce indicator variables for N and T such that P is the reference category; indicator variables for cycles such that cycle 1 is the reference category; and an indicator variable for the arm such that X = 1 represents the intervention and X = 0 placebo. The saturated model can then be expressed mathematically as


$$\log\left[\frac{P\left(y_{its}\geq k\right)}{1-P\left(y_{its}\geq k\right)}\right]\;=\alpha_{k}+\beta_{1}N+\beta_{2}T+\beta_{3}T2+\beta_{4}T3+\beta_{5}T4+\beta_{6}T5+\beta_{7}T6+\beta_{8}T7+\beta_{9}N*T2\;+\beta_{10}N*T3+\beta_{11}N*T4+\beta_{12}N*T5+\beta_{13}N*T6+\beta_{14}N*T7+\beta_{15}T*T2\;+\beta_{16}T*T3+\beta_{17}T*T4+\beta_{18}T*T5+\beta_{19}T*T6+\beta_{20}T*T7+\beta_{21}\;X+\beta_{22}X\;*N+\beta_{23}X*T+\beta_{24}X*T2+\beta_{25}X*T3+\beta_{26}X*T4+\beta_{27}X*T5\;\;\;\;\;\;\;+\beta_{28}X\;*T6+\beta_{29}X*T7+\beta_{30}X*N*T2+\beta_{31}X*N*T3+\beta_{32}X*N*T4+\beta_{33}X*N\;*T5\;\;+\beta_{34}X*N*T6+\beta_{35}X*N*T7+\beta_{36}X*T*T2+\beta_{37}X*T*T3\;+\beta_{38}X*T*T4+\beta_{39}X*T*T5+\beta_{40}X*T*T6+\beta_{41}X*T*T7+u_{is},\;\;\;\;\;\;\;\;k\;=2,\;3.$$

Because there are two cumulative logits (K−1=2), the model has two intercepts, $\alpha_{2}$ and $\alpha_{3}$. Because of randomization, it is reasonable to assume that all patients have the same scores at baseline for all three symptoms; therefore, we assume the parameters $\beta_{21}=\beta_{22}=\beta_{23}=0$. The 18 parameters from $\beta_{24}$ to $\beta_{41}$ allow the patterns of change from baseline of the odds of being greater than or equal to a particular response category for N, T and P not to be the same in the two arms (i.e., these parameters represent the arm x time interaction). The null hypothesis of no arm x time interaction versus the alternative hypothesis can then be expressed as


$$H_{0}:\;\beta_{24}=\beta_{25}=\beta_{26}=\beta_{27}=\beta_{28}=\beta_{29}=\beta_{30}=\beta_{31}=\beta_{32}=\beta_{33}=\beta_{34}=\beta_{35}=\beta_{36}=\beta_{37}=\beta_{38}\;=\beta_{39}=\beta_{40}=\beta_{41}=0.\;\mathrm{versus}\;H_{a}:\mathrm{At}\;\mathrm{least}\;\mathrm{one}\;\mathrm{parameter}\;\mathrm{is}\;\mathrm{not}\;\mathrm{equal}\;\mathrm{to}\;\mathrm{zero.}$$

We compare the Wald statistic to a $\chi^{2}$ distribution with 18 degrees of freedom, which equals the number of parameters being tested.

#### 4.1.3. Scenarios and Simulating Power

**Figure 3:**
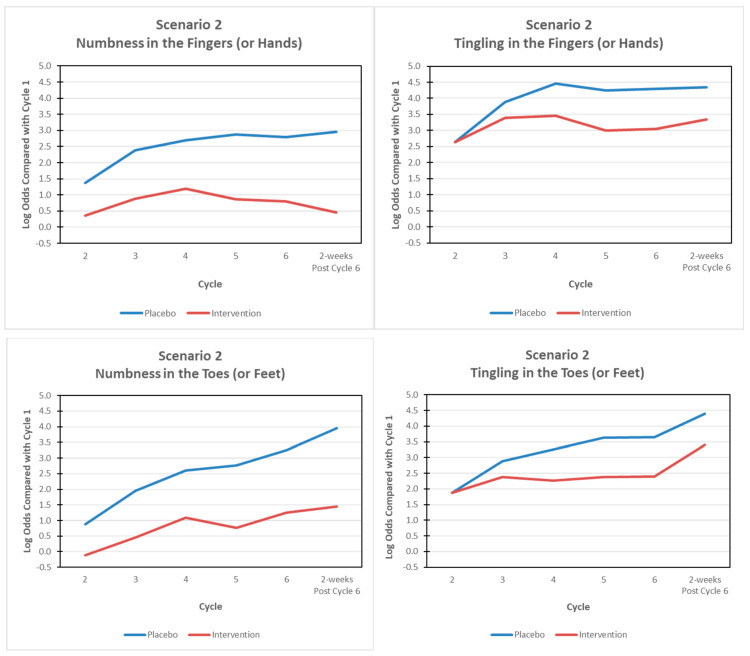
Design Application. **Scenario 2:** An arm-by-treatment interaction such that a large effect for numbness, a moderate effect for tingling and no effect for shooting/burning pain was assumed. The log odds compared with cycle 1 (baseline) for the placebo arm (blue) were based on the analysis of the pooled placebo arms from two recently completed OIPN prevention trials (NCCTG N08CB; MC11C4).

Two scenarios were considered. In scenario 1, we assumed no arm-by-time interaction (i.e., responses over time coincide) such that the log odds compared with cycle 1 (baseline) were the same in each arm and corresponded to the log odds obtained from our data analysis of the pooled placebo arms (NCCTG N08CB; MC11C4). In scenario 2, we hypothesized an arm-by-time interaction such that a large effect for N, a moderate effect for T and no effect for P was assumed. Figure 3 graphically shows the assumed effects in scenario 2 for N and T in the fingers (or hands) and toes (or feet). In this illustration, we sought to randomize 100 patients (50 per arm). Power calculations were obtained via simulation. In total, 1000 datasets were generated from each model. We assumed a two-sided significance level of α=0.05 for both tests such that there was no adjustment for multiplicity. The proportion of times we rejected the null hypothesis, or equivalently the power, was recorded separately for the fingers (or hands) and toes (or feet). For each symptom and at each two-week cycle (2, 3, 4, 5 and 6), as well as at 2 weeks post-cycle 6, we report the estimated average log odds (compared with cycle 1, baseline) over the 1000 datasets generated as a measure of the true estimate and the corresponding empirical standard error. Simulating power was conducted using SAS.

### 4.2. Results

The results from generating 1000 datasets under scenario 1 are shown in Table 2A,B for the fingers (or hands) and toes (or feet), respectively. The proportion of times we rejected $H_{0}:$ No arm x time interaction was 5.2% and 4.9% for the fingers (or hands) and toes (or feet), respectively. Because we generated the data assuming no arm-by-time interaction in scenario 1, we were able to reasonably recover the Type I error of 5%.

The results from generating 1000 datasets under scenario 2 are shown in Table 3A,B for the fingers (or hands) and toes (or feet), respectively. The proportion of times we rejected $H_{0}:$ No arm x time interaction was 98.1% and 94.8% for the fingers (or hands) and toes (or feet), respectively. In summary, testing at the 5% significance level, if we randomized 100 patients in a 1:1 fashion to either the intervention or placebo, the study would have 98.1% and 94.8% power to detect the stated interaction effects for the fingers (or hands) and toes (or feet), respectively.

## 5. Discussion

The results of this evaluation demonstrate that, despite a shorter duration of oxaliplatin (3 months instead of 6 months), OIPN was still a pervasive problem, with patients experiencing considerable mild to moderate N and T in the lower and upper distal extremities. To avoid the debilitating sequelae from OIPN [9,10] and to ensure that patients continue to receive the most efficacious doses of oxaliplatin, identification of effective OPIN preventative interventions is still needed, regardless of whether oxaliplatin is planned to be given for 3 months or 6 months.

To our knowledge, this is the first time that the likelihood and corresponding uncertainty of the estimated likelihood of symptom-specific timing, location and severity of patient-reported responses over 3 months (6 cycles) of oxaliplatin was formally quantified and is reported in the literature. In addition to providing patients and clinicians with an accurate quantification of the natural history of OIPN specifically germane to the adoption of a shorter 3-month duration of oxaliplatin, these population-averaged probability estimates (averaged over patients), which were obtained from a large cohort of patients who received a placebo, can directly inform the design of a subsequent, hypothesis-driven, placebo-controlled OIPN trial. Specifically, these population-averaged probabilities represent the natural history of the symptom-specific timing, location and severity of patient-reported responses over 3 months of oxaliplatin that would be expected on the placebo arm of a subsequent trial. We demonstrated, by way of illustration, how to express and operationalize the scientifically interesting hypothesis for an arm-by-time interaction and illustrated how to simulate statistical power to compare two arms in terms of their patterns of change from baseline in the odds of being greater than or equal to a particular response category for N, T and P separately in the upper/lower extremities. Adopting such an approach would ensure that the study design is compatible with the analytic method used in the primary analysis. The SAS programs developed for this paper to simulate power can be made available from the corresponding author on request.

Designing and analyzing data from a placebo-controlled OIPN symptom intervention trial based on an overall test of the arm-by-time interaction is appealing for several reasons. First, in such a trial, we are primarily interested in testing the hypotheses that compare the intervention and the placebo in terms of changes in the responses over time. In a randomized study, baseline symptoms are expected to be similar between arms. Failing to reject the null hypothesis (same pattern of change over time between the arms) necessarily means that the responses over time coincide. Second, the overall test of the arm-by-time interaction is completely general. The overall test does not target any specific pattern for the difference in responses over time between the arms. If the difference between arms takes a form different from the pattern hypothesized as part of the study design, one can still achieve statistical significance based on the overall test for an arm-by-time interaction. This would not be true, for example, if the study was designed by choosing a within-patient change from baseline endpoint, say, based on a total sensory neuropathy score, at a single point in time (e.g., 2 weeks post-cycle 6). The added sensitivity with such a specific endpoint comes with a price. If the study fails to detect a between-arm difference in the change from baseline to two weeks post-cycle 6, which would have been chosen in advance, the overall test for an arm-by-time interaction can still obtain a statistically significant result due to its generality. Third, while the overall test of interaction does not indicate how the two arms differ, it is straightforward to examine the regression coefficients from the fitted model and their standard errors to ascertain where the differences lie over time and according to which symptom (N, T and P).

There is no accepted primary endpoint in the design and analysis of randomized clinical trials evaluating OIPN in cancer patients receiving oxaliplatin-based chemotherapy [11]. In our opinion, it is appealing to define the primary endpoint as the serially measured EORTC QLQ-CIPN20 sensory scores for N, T and P in upper/lower extremities self-reported by the patient on day 1 of each cycle prior to oxaliplatin treatment (cycles 1–6) and 2 weeks after the 6th cycle and then adopt our statistical modeling approach that appreciates the nuanced and complex clinical course of OIPN that presents with substantial patient heterogeneity. The QLQ-CIPN20 was developed by the EORTC to assess CIPN [12]. The EORTC QLQ-CIPN20 is a multidimensional tool that has been well validated and captures the broad scope of the symptom experience; the tool recognizes that patient-reported outcomes are better tools for measuring symptoms than are physician-determined means. The statistical model we used to analyze such an endpoint was compatible with the complex clinical course of OIPN and could quantify the likelihood of symptom-specific timing, location and severity of patient-reported responses of N, T and pain in the upper and lower distal extremities. In addition, the proposed modeling approach could adequately handle missing data (intermittent and due to drop out) by incorporating the patients’ serial oxaliplatin doses, which are highly correlated with the response, into the imputation model to improve the imputations; missing data are a major issue in data analysis—25% of patients discontinue oxaliplatin therapy given for 6 months (12 cycles) and, in two-thirds of these patients, the reason for discontinuation is due to OIPN symptoms [5]. Lastly, we show how such a seemingly complex model can be straightforwardly used to design a hypothesized-driven phase III clinical trial to meaningfully compare a novel intervention to placebo such that the statistical design is compatible with the statistical analysis.

Substantial patient heterogeneity in the likelihood of a more severe response for a symptom was observed and the degree of heterogeneity was quite different between each symptom in the fingers (or hands), with the largest degree of heterogeneity seen with P. We do not fully understand why this variation occurs. Suspected reasons vary widely in the literature and the reasons are likely multifactorial and may include genetic factors, certain comorbidities and the mechanism of nerve injury. Without clear understanding of patient-level factors affecting OIPN, we were reluctant to adjust for them in the statistical models; rather, we accounted for symptom heterogeneity across patients by including random effects for each symptom in the models. Another limitation of our research study is that there may be other analytic approaches different from the analytic approach adopted in this article which may be better at detecting intervention effects in OIPN trials; therefore, future research is needed to compare the performance of different analytic approaches.

## 6. Conclusions

While previous reports described patients’ experience with OIPN planned to be given for 6 months (12 cycles), this paper provided a more accurate quantification of a patient’s treatment experience with shorter (3 months/6 cycles) oxaliplatin treatment. Distinct patterns in the probabilities for each response over time for N and T were observed between the upper/lower distal extremities, while the probabilities of a response >1 for P was largely negligible in both locations. Despite the decrease in exposure to oxaliplatin from 6 to 3 months, OIPN is still pervasive with patients experiencing considerable N and T in the fingers (or hands) and toes (or feet). Identification of effective OPIN preventative interventions is still needed when oxaliplatin is planned to be given for the shorter, 3-month duration (6 cycles). We illustrated how the probability estimates for each response over time derived in this article can be used to design subsequent placebo-controlled OIPN trials.

## Figures and Tables

**Table 1:** (**A**) Format of the ordinal data in the fingers (or hands) for a single patient. (**B**) Summary of notation used in Model 1 and Model 2.

(A)				
Patient (*i*)	Cycle (*t*)	Symptoms (*s*)		
		Fingers (or Hands)		
		Numbness	Tingling	Pain
1001	1	$y_{111}$	$y_{112}$	$y_{113}$
	2	$y_{121}$	$y_{122}$	$y_{123}$
	3	$y_{131}$	$y_{132}$	$y_{133}$
	4	$y_{141}$	$y_{142}$	$y_{143}$
	5	$y_{151}$	$y_{152}$	$y_{153}$
	6	$y_{161}$	$y_{162}$	$y_{163}$
	7	$y_{171}$	$y_{172}$	$y_{173}$
**(B)**				
**Notation**	**Description**	**Possible Values**	**Notes**	
$y_{its}$	Response for patient i at cycle t and symptom s	1 = “Not at all”, 2 = “A little”, 3 = “Quite a bit”, 4 = “Very much”	Due to data sparsity, response outcomes of 3–4 (“Quite a bit” and “Very much”) were combined such that there were K=3 response categories and K−1=2 cumulative logits	
i	Index for patient	1, 2, …, *n*		
t	Day 1 of each 2-week cycle prior to oxaliplatin treatment (cycles 1–6), as well as 2 weeks after the 6th cycle (day 1 of cycle 7, prior to oxaliplatin treatment)	1, 2, 3, 4, 5, 6, 7	Indicator variables for cycles T2 = *I*(*t* = 2), T3 = *I*(*t* = 3), T4 = *I*(*t* = 4), T5 = *I*(*t* = 5), T6 = *I*(*t* = 6) and T7 = *I*(*t* = 7; two weeks post-cycle 6), such that cycle 1 (*t* = 1) was the reference category	
s	Symptom	1 = N (numbness), 2 = T (tingling), 3 = P (shooting/burning pain)	Indicator variables for N = *I*(*s* = 1) and T = *I*(*s* = 2), such that P (*s* = 3) was the reference category	
$\left(u_{i1},\;u_{i2},u_{i3}\right)$	Multivariate random effect that describes patient heterogeneity for N, T and P		Assumed that the set $\left\{\left(u_{i1},\;u_{i2},u_{i3}\right)\right\}$ was independent from a multivariate normal distribution, $N\left(0,\;\Sigma \right)$, with possibly different variances and nonzero correlations	
$\sigma_{1}^{2}$, $\sigma_{2}^{2}$, $\sigma_{3}^{2}$	Variance component $\sigma_{1}^{2}$ for $\left\{u_{i1}\right\}$, $\sigma_{2}^{2}$ for $\left\{u_{i2}\right\}$ and $\sigma_{3}^{2}$ for $\left\{u_{i3}\right\}$		The larger was $\sigma_{1}^{2}$, $\sigma_{2}^{2}$, or $\sigma_{3}^{2}$, the more heterogeneous was the likelihood at a given cycle of a more severe response for that symptom across patients	
$\rho_{12}$, $\rho_{13}$, $\rho_{23}$	The correlations between the random effects were $\rho_{12}$ for $\left\{u_{i1},\;u_{i2}\right\}$, $\rho_{13}$ for $\left\{u_{i1},\;u_{i3}\right\}$ and $\rho_{23}$ for $\left\{u_{i2},\;u_{i3}\right\}$		Allowed the random effects for each symptom to be correlated and the degree of correlation to be different	

Note. Here, $y_{its}$ represents the response at cycle *t* on symptom *s* for patient *i* (summary of notation is further detailed in Table 1B). The format of the ordinal data in the toes (or feet) is the same.

**Table 2:** Design application. **Scenario 1:** For each log odds (compared with cycle 1, baseline), we report the average estimate over the 1000 datasets generated as a measure of the true estimate and the corresponding standard deviation (SD) or empirical standard error. The “true” estimates of the log odds compared with cycle 1 (baseline) for the placebo arm were based on the analysis of the pooled placebo arms from two recently completed OIPN prevention trials (NCCTG N08CB; MC11C4). The “true” estimates of the log odds compared with cycle 1 (baseline) assumed for the treatment arm were hypothesized as being the same as the placebo arm (i.e., responses over time coincide). Note that cycle 1 served as a baseline (prior to oxaliplatin exposure), while patient-reported responses measured at each two-week cycle (2–6), as well as at 2 weeks post-cycle 6 (day 1 of cycle 7, prior to oxaliplatin treatment), served as the post-baseline measurements.

A. Fingers (or Hands)					
Symptom	Cycle	Placebo Log Odds		Treatment Log Odds	
		Truth	Average (SD)	Truth	Average (SD)
Numbness	2	1.3660	1.39 (0.57)	1.3660	1.40 (0.59)
Numbness	3	2.3890	2.45 (0.55)	2.3890	2.47 (0.56)
Numbness	4	2.6887	2.75 (0.55)	2.6887	2.78 (0.56)
Numbness	5	2.8689	2.95 (0.54)	2.8689	2.94 (0.55)
Numbness	6	2.7988	2.86 (0.54)	2.7988	2.87 (0.55)
Numbness	7	2.9552	3.02 (0.53)	2.9552	3.04 (0.55)
Pain	2	2.5269	2.62 (0.98)	2.5269	2.65 (1.01)
Pain	3	4.0743	4.24 (0.94)	4.0743	4.24 (0.94)
Pain	4	3.7259	3.87 (0.94)	3.7259	3.89 (0.94)
Pain	5	3.6576	3.78 (0.95)	3.6576	3.80 (0.94)
Pain	6	3.6052	3.76 (0.94)	3.6052	3.75 (0.94)
Pain	7	3.3041	3.43 (0.95)	3.3041	3.43 (0.95)
Tingling	2	2.6389	2.68 (0.51)	2.6389	2.68 (0.52)
Tingling	3	3.8843	3.96 (0.52)	3.8843	3.94 (0.51)
Tingling	4	4.4525	4.52 (0.52)	4.4525	4.52 (0.53)
Tingling	5	4.2422	4.33 (0.52)	4.2422	4.32 (0.51)
Tingling	6	4.2994	4.37 (0.53)	4.2994	4.37 (0.51)
Tingling	7	4.3483	4.44 (0.51)	4.3483	4.41 (0.53)
**B. Toes (or Feet)**					
**Symptom**	**Cycle**	**Placebo Log Odds**		**Treatment Log Odds**	
		**Truth**	**Average (SD)**	**Truth**	**Average (SD)**
Numbness	2	0.8810	0.81 (0.80)	0.8810	0.93 (0.77)
Numbness	3	1.9507	1.95 (0.73)	1.9507	2.01 (0.72)
Numbness	4	2.5956	2.64 (0.68)	2.5956	2.69 (0.69)
Numbness	5	2.7656	2.84 (0.69)	2.7656	2.86 (0.68)
Numbness	6	3.2492	3.33 (0.66)	3.2492	3.35 (0.69)
Numbness	7	3.9473	4.04 (0.66)	3.9473	4.06 (0.65)
Pain	2	1.1690	1.19 (0.97)	1.1690	1.23 (0.97)
Pain	3	2.1083	2.18 (0.88)	2.1083	2.21 (0.96)
Pain	4	2.5705	2.67 (0.89)	2.5705	2.68 (0.89)
Pain	5	2.1911	2.27 (0.90)	2.1911	2.27 (0.89)
Pain	6	2.9370	3.03 (0.85)	2.9370	3.09 (0.87)
Pain	7	3.0698	3.19 (0.85)	3.0698	3.23 (0.83)
Tingling	2	1.8795	1.93 (0.67)	1.8795	1.96 (0.66)
Tingling	3	2.8853	2.97 (0.61)	2.8853	3.01 (0.63)
Tingling	4	3.2611	3.37 (0.63)	3.2611	3.39 (0.66)
Tingling	5	3.6370	3.75 (0.63)	3.6370	3.77 (0.61)
Tingling	6	3.6487	3.77 (0.62)	3.6487	3.84 (0.61)
Tingling	7	4.4045	4.54 (0.62)	4.4045	4.57 (0.65)

**Table 3:** Design application. **Scenario 2:** For each log odds (compared with cycle 1, baseline), we report the average estimate over the 1000 datasets generated as a measure of the true estimate and the corresponding standard deviation (SD) or empirical standard error. The “true” estimates of the log odds compared with cycle 1 (baseline) for the placebo arm were based on the analysis of the pooled placebo arms from two recently completed OIPN prevention trials (NCCTG N08CB; MC11C4). The “true” estimates of the log odds compared with cycle 1 (baseline) assumed for the treatment arm were hypothesized consistent with an arm-by-time interaction such that a large effect for numbness, a moderate effect for tingling and no effect for pain was assumed. Note that cycle 1 served as a baseline (prior to oxaliplatin exposure), while patient-reported responses measured at each two-week cycle (2–6), as well as at 2 weeks post-cycle 6 (day 1 of cycle 7, prior to oxaliplatin treatment), served as the post-baseline measurements.

A. Fingers (or Hands)					
Symptom	Cycle	Placebo Log Odds		Treatment Log Odds	
		Truth	Average (SD)	Truth	Average (SD)
Numbness	2	1.3660	1.42 (0.57)	0.3660	0.35 (0.63)
Numbness	3	2.3890	2.46 (0.56)	0.8890	0.89 (0.60)
Numbness	4	2.6887	2.75 (0.55)	1.1887	1.18 (0.59)
Numbness	5	2.8689	2.92 (0.54)	0.8689	0.85 (0.62)
Numbness	6	2.7988	2.86 (0.56)	0.7988	0.79 (0.62)
Numbness	7	2.9552	3.03 (0.57)	0.4552	0.43 (0.64)
Pain	2	2.5269	2.65 (1.04)	2.5269	2.55 (1.04)
Pain	3	4.0743	4.18 (1.02)	4.0743	4.22 (0.96)
Pain	4	3.7259	3.82 (1.03)	3.7259	3.85 (0.99)
Pain	5	3.6576	3.76 (1.01)	3.6576	3.77 (1.00)
Pain	6	3.6052	3.70 (1.02)	3.6052	3.69 (1.00)
Pain	7	3.3041	3.36 (1.06)	3.3041	3.41 (1.03)
Tingling	2	2.6389	2.70 (0.50)	2.6389	2.70 (0.49)
Tingling	3	3.8843	3.96 (0.51)	3.8843	3.45 (0.50)
Tingling	4	4.4525	4.54 (0.53)	3.4525	3.52 (0.52)
Tingling	5	4.2422	4.33 (0.52)	2.9922	3.06 (0.49)
Tingling	6	4.2994	4.38 (0.53)	3.0494	3.12 (0.51)
Tingling	7	4.3483	4.43 (0.53)	3.3488	3.41 (0.50)
**B. Toes (or Feet)**					
**Symptom**	**Cycle**	**Placebo Log Odds**		**Treatment Log Odds**	
		**Truth**	**Average (SD)**	**Truth**	**Average (SD)**
Numbness	2	0.8810	0.87 (0.79)	-0.1190	-0.19 (0.92)
Numbness	3	1.9507	1.99 (0.72)	0.4507	0.44 (0.83)
Numbness	4	2.5956	2.70 (0.69)	1.0956	1.13 (0.77)
Numbness	5	2.7656	2.85 (0.68)	0.7656	0.79 (0.78)
Numbness	6	3.2492	3.37 (0.65)	1.2492	1.30 (0.76)
Numbness	7	3.9473	4.09 (0.67)	1.4473	1.49 (0.74)
Pain	2	1.1690	1.24 (0.97)	1.1690	1.17 (1.02)
Pain	3	2.1083	2.15 (0.93)	2.1083	2.18 (0.89)
Pain	4	2.5705	2.64 (0.89)	2.5705	2.63 (0.88)
Pain	5	2.1911	2.30 (0.91)	2.1911	2.30 (0.92)
Pain	6	2.9370	3.06 (0.87)	2.9370	3.06 (0.84)
Pain	7	3.0698	3.21 (0.85)	3.0698	3.22 (0.84)
Tingling	2	1.8795	1.95 (0.69)	1.8795	1.93 (0.67)
Tingling	3	2.8853	2.99 (0.67)	2.3853	2.48 (0.65)
Tingling	4	3.2611	3.38 (0.67)	2.2611	2.36 (0.65)
Tingling	5	3.6370	3.77 (0.65)	2.3870	2.51 (0.65)
Tingling	6	3.6487	3.77 (0.65)	2.3987	2.50 (0.65)
Tingling	7	4.4045	4.56 (0.67)	3.4045	3.55 (0.63)

## Data Availability

All data presented from the two clinical trials involved in this report are available on request from the corresponding author.

## Supplementary Materials

The following supporting information can be downloaded at: https://www.mdpi.com/article/10.3390/cancers14051212/s1, Table S1: EORTC QLQ-CIPN20—The first 6 questions (Q31–36) correspond to numbness, tingling, and shooting/burning pain in the upper/lower distal extremities.
