# Supplementary material for: Temporal, Location- and Symptom-Specific Likelihood of Patient-Reported Sensory Symptoms Related to Oxaliplatin-Induced Peripheral Neuropathy (OIPN) in Patients Receiving Oxaliplatin for Three Months
Source: Cancers (Basel). 2022 Feb 25;14(5):1212. doi: 10.3390/cancers14051212 (PMC8909760; doi:10.3390/cancers14051212)
Supplement: Supplementary file 1 [file cancers-14-01212-s001.zip › cancers-1577556 -supplementary.pdf]

cancers

Table S1. EORTC QLQ-CIPN20—The first 6 questions (Q31–36) correspond to numbness, tingling, and shooting/burning pain in the upper/lower distal extremities.

EORTC QLQ – CIPN20

Patients sometimes report that they have the following symptoms or problems. Please indicate the extent to which you have experienced these symptoms or problems during the past week. Please answer by circling the number that best applies to you.

During the past week :	Not at All	A Little	Quite a Bit	Very Much
31 Did you have tingling fingers or hands?	1	2	3	4
32 Did you have tingling toes or feet?	1	2	3	4
33 Did you have numbness in your fingers or hands?	1	2	3	4
34 Did you have numbness in your toes or feet?	1	2	3	4
35 Did you have shooting or burning pain in your fingers or hands?	1	2	3	4
36 Did you have shooting or burning pain in your toes or feet?	1	2	3	4
37 Did you have cramps in your hands?	1	2	3	4
38 Did you have cramps in your feet?	1	2	3	4
39 Did you have problems standing or walking becauseof difficulty feeling the ground under your feet?	1	2	3	4
40 Did you have difficulty distinguishingbetween hot and cold water?	1	2	3	4
41 Did you have a problem holding a pen, whichmade writing difficult?	1	2	3	4
42 Did you have difficulty manipulating small objects withyour fingers (for example, fastening small buttons)?	1	2	3	4
43 Did you have difficulty opening a jar or bottle because of weakness in your hands?	1	2	3	4
44 Did you have difficulty walking because yourfeet dropped downwards?	1	2	3	4
45 Did you have difficulty climbing stairs or getting upout of a chair because of weakness in your legs?	1	2	3	4
46 Were you dizzy when standing up froma sitting or lying position?	1	2	3	4
47 Did you have blurred vision?	1	2	3	4
48 Did you have difficulty hearing?	1	2	3	4
**Please answer the following question only if you drive a car**				
49 Did you have difficulty using the pedals?	1	2	3	4
**Please answer the following question only if you are a man**				
50 Did you have difficulty getting or maintaining an erection?	1	2	3	4

© QLQ-CIPN20 Copyright 2003 EORTC Quality of life Group. All rights reserved.
